# High-dimensional single-cell analyses reveal neutrophil heterogeneity in guttate psoriasis

**DOI:** 10.1016/j.ebiom.2026.106172

**Published:** 2026-02-19

**Authors:** Avinash Padhi, Anoop T. Ambikan, Panagiotis Andriopoulos, Indranil Sinha, Mira Akber, Wenning Zheng, Rokeya Sultana Rekha, Laura Palma Medina, Jan-Inge Henter, Mattias Svensson, Liv Eidsmo, Anna Norrby-Teglund, Ujjwal Neogi, Peter Bergman, Josefin Lysell, Magda Lourda

**Affiliations:** a Center for Infectious Medicine, Department of Medicine Huddinge, Karolinska Institutet, Karolinska University Hospital, Stockholm, Sweden; b Division of Dermatology and Venereology, Department of Medicine Solna, Karolinska Institutet, Stockholm, Sweden; c The Systems Virology Laboratory, Division of Clinical Microbiology, Department of Laboratory Medicine, Karolinska Institutet, Stockholm, Sweden; d LEO Foundation Skin Immunology Research Center, Department of Microbiology and Immunology, Copenhagen University, Copenhagen, Denmark; e Division of Clinical Immunology, Department of Laboratory Medicine, Karolinska Institutet, Stockholm, Sweden; f Childhood Cancer Research Unit, Department of Women's and Children's Health, Karolinska Institutet, Stockholm, Sweden; g Astrid Lindgren Children's Hospital, Karolinska University Hospital, Stockholm, Sweden; h PO Rheumatology/Dermatology/Gastroenterology, Karolinska University Hospital, Stockholm, Sweden; i Department of Clinical Immunology and Transfusion Medicine, Karolinska University Hospital, Stockholm, Sweden

**Keywords:** Antigen-presenting neutrophils, Group A Streptococcus (GAS), Guttate psoriasis (GP), Neutrophils, Psoriatic inflammation

## Abstract

**Background:**

Guttate psoriasis (GP) is associated with streptococcal throat infection. Neutrophils are the first immune cells to respond to Group A Streptococcal (GAS) infection, but detailed analysis of their contribution to GP pathogenesis is lacking. The study primarily focuses on phenotyping the neutrophils located at the site of inflammation in GP skin and understanding their specific contribution to the pathogenesis of the disease.

**Methods:**

Here, we performed a comprehensive immunophenotypic and transcriptomic analysis of neutrophils from blood and inflamed GP skin using high-dimensional single-cell protein and RNA analyses. *Ex vivo* stimulation of neutrophils and co-culture with CD4^+^ T cells was performed to validate the function of neutrophil subsets upon GAS stimulation.

**Findings:**

We uncovered high diversity of human neutrophils in GP, with enrichment of an immature subset in GP skin that exhibited antigen processing and presentation signature. A similar subset in healthy controls was observed only upon *ex vivo* stimulation of neutrophils with GAS bacteria. Additionally, the GAS-induced neutrophil subset was found to induce CD4^+^ T cell proliferation mediated by HLA-DR. Other neutrophil subsets that expanded in GP were characterised by enrichment of genes related to neutrophil migration, formation of neutrophil extracellular traps and phagocytosis.

**Interpretation:**

Our study depicts the landscape of neutrophils in GP skin and highlights HLA-DR^+^ neutrophils with possible role in disease pathogenesis.

**Funding:**

This work was supported by 10.13039/501100022654HudFonden, Psoriasisfonden, Gösta A Karlssons 60-års fond, Magnus Bergvall stiftelse, Åke Wibergs stiftelse, and 10.13039/501100004047Karolinska Institute. AP was supported by 10.13039/501100022654HudFonden. PA was partially supported by Karolinska Institute’s doctoral funding (KID). LPM was supported by grants from 10.13039/501100003748Svenska Sällskapet för Medicinsk Forskning (SSMF) and 10.13039/501100001704European Society of Clinical Microbiology and Infectious Diseases (ESCMID). UN was supported by grants from the 10.13039/501100004359Swedish Research Council. JL was supported by Region Stockholm (clinical postdoctoral appointment). ML was supported by the Swedish Childhood Cancer research fund and the 10.13039/501100004047Karolinska Institute. Part of the data handling was enabled by resources at project number SNIC-2021/22-49 provided by the Swedish National Infrastructure for Computing (SNIC), partially funded by the 10.13039/501100004359Swedish Research Council through grant agreement no. 2018-05973.

**Research in context**

**Evidence before this study**

Guttate psoriasis (GP) is an inflammatory skin disease where small psoriasiform plaques rapidly appear over the whole body, often in response to streptococcal throat infection. Neutrophils are the first immune cells to respond to Group A Streptococcal (GAS) infection, but a detailed analysis of human neutrophils in GP inflammation is lacking.

**Added value of this study**

Our study combined single-cell RNA sequencing (scRNA-seq) and multicolour flow cytometry to immunophenotype cells from skin and peripheral blood. We found high diversity amongst circulating and extravasated neutrophils in GP, with enrichment of a subset of antigen-presenting neutrophils in skin. Through an *ex vivo* stimulation assay we showed that GAS stimulation of circulating neutrophils drove enrichment of antigen-presenting neutrophils. These GAS-stimulated neutrophils could induce proliferation of autologous CD4^+^ T cells through HLA-DR. This provides a mechanistic scenario for the rapid spread of neutrophil extravasation in GP during GAS infection.

**Implications of all the available evidence**

By establishing a connection between GAS infection and the increased presence of antigen-presenting neutrophils in GP skin, we propose that the rapid dissemination of circulating, GAS-activated neutrophils enhances antigen presentation. Antigen-presenting neutrophils add another potential “route” for T cell activation in skin following streptococcal infection, presenting a promising target for early intervention to halt T cell activation during progressive GP.

## Introduction

Guttate psoriasis (GP), a distinct form of acute onset psoriasis, is an inflammatory skin disease characterised by the sudden appearance of red, scaly, and smaller skin lesions widespread over the body. GP typically follows an infection, most commonly tonsillitis caused by Group A Streptococcus (GAS), and usually affects adolescents and young adults.^1^ Several theories on how GAS triggers GP have been proposed, including molecular mimicry between Streptococcal M-protein and keratins in the skin as well as immune activation through superantigens, but no definite mechanism has been established.^2^^,^^3^ In approximately one third of patients, GP develops into chronic plaque psoriasis, which can also flare upon GAS infection.^4^ In individuals with genetic susceptibility, an association between an environmental trigger, such as an upper respiratory tract infection, and the onset of an inflammatory condition in a distant organ has been shown for numerous inflammatory diseases, including skin, rheumatic, heart and kidney disorders.^5^ However, the molecular links between an infectious trigger and activation of inflammatory diseases are not well understood.

Upon infection, GAS colonises and invades the tonsillar epithelium leading to secretion of inflammatory mediators by epithelial cells, neutrophils, macrophages, and dendritic cells (DCs).^6^ Recognition by DCs promotes maturation of Th1 cells, while macrophages and epithelial cells promote maturation of Th17 cells.^7^^,^^8^ Notably, *TCRVB* gene rearrangement in cutaneous and tonsillar T cells isolated from the same patient demonstrates that T cells from the tonsils later migrate to the skin.^9^ Once in the skin, incoming T cells, predominantly with a Th17 phenotype, induce an inflammatory response that initiates psoriatic plaque formation.^10^ It has been established that IL-17A produced by Th17 cells promotes neutrophil migration and activation, while neutrophils produce IL-17A and IL-23 that in turn attract Th17 cells, resulting in a vicious circle of inflammation.^11^^,^^12^ A similar loop exists between neutrophils and keratinocytes in psoriasis.^13^

Neutrophils accumulate in psoriatic skin, forming the pustules of Kogoj and Munro microabscesses in the *Stratum corneum*.^14^ Once in the skin, they sustain local inflammation by release of antimicrobial peptides (AMPs) through degranulation, production of reactive oxygen species (ROS) and formation of neutrophil extracellular traps (NETs).^15^ Recently, neutrophils were shown to present antigens to T cells via MHC-II,^16^, ^17^, ^18^, ^19^ stimulate T cell proliferation in response to superantigens^20^ and increase DC maturation.^21^ The multifaceted role played by neutrophils underscores the presence of distinct subpopulations with specialised tasks. Heterogeneous populations of neutrophils have been described in health and disease based on cell-surface markers, maturity, functions, localisation, and transcriptional diversity.^22^, ^23^, ^24^, ^25^ In healthy individuals, most neutrophils have a mature CD16^bright^ phenotype, while immature neutrophils (CD16^dim^) comprise <1% of total neutrophils in circulation. In inflammatory conditions, diverse neutrophil phenotypes have been observed, such as increased frequency of both immature and mature activated neutrophil subsets in different patient groups.^25^, ^26^, ^27^, ^28^ In addition, the local tissue microenvironment also dictates the diversity amongst neutrophils.^29^ Over time, neutrophils develop an aged phenotype characterised by increased expression of CXCR4.^30^ In psoriatic skin, unique neutrophil subsets have been identified, including the ones that respond to targeted therapy.^29^ However, most of the existing studies focus on circulating neutrophils, and very few describe the populations at the site of inflammation. Thus, a systematic analysis of the phenotypic and transcriptomic changes occurring in human neutrophils during GP is required for the interpretation and targeting of these cells.

In the present study, we set out to perform a comprehensive immunophenotyping and transcriptome analysis at the single-cell level of human neutrophils in blood and lesional skin samples from patients with GP. In addition, we explored the mechanisms by which neutrophils could interact with other immune cells and performed *ex vivo* functional studies to determine their possible roles. This revealed a complex neutrophil landscape in GP skin with enrichment of neutrophils with an antigen-presenting phenotype.

## Methods

### Study design and sample collection

Written informed consent was obtained from all patients before enrolment. Inclusion criteria were established prospectively, including a definite diagnosis of GP by a senior consultant dermatologist (JL) and no topical treatment for skin lesions two weeks prior to inclusion. Punch biopsies (3 × 4 mm full thickness) were obtained from lesional, affected skin from the patients. All patients self-identified as White of Northern European ancestry. Sex was self-reported by participants and was concordant with clinical records. Sex was not considered in the study design or analysis. Detailed clinical characteristics of the patients as well as information on the experiments each sample was used for are provided in Table 1. Blood samples from healthy controls (n = 40) were collected from healthy volunteers in the Department of Dermatology or from the Clinical Immunology and Transfusion Medicine laboratory in Karolinska University Hospital.

**Table 1:** Characteristics of the patients with GP included in the study.

Patient	Age (years)	Sex	Additional disease	Medication at sampling	Guttate episode previously	Plaque psoriasis previously	Verified GAS throat infection up to 3 months prior to inclusion	PASI	Experiment
1	65	F	None	None	No	Yes, very mild	Yes	6.8	FC
2	21	F	None	None	Yes	No	No^a^	8	FC
3	25	F	Depression	SSRI	No	No	Yes	3	FC
4	39	F	IBD	6-MP	No	No	Yes	6.8	FC
5	35	F	None	None	No	Yes, very mild	Yes	14	FC
6	44	M	None	Penicillin V	No	Yes, very mild	Yes	7	FC
7	44	M	None	None	No	No	Yes	5.8	FC/Sorting/10X/IF
8	19	F	None	None	Yes	No	Yes	4	FC/Sorting/10X
9	25	F	Henoch-Schönlein purpura 6 years prior to inclusion	None	No	Yes, very mild	Yes	9.8	FC/Sorting/10X
10	35	M	Anxiety disorder	SSRI	No	Yes, very mild	Yes	4.6	FC/Sorting/10X
11	43	M	None	None	Yes	No	Yes	4	FC
12	46	F	None	None	Yes	No	Yes	8.2	FC
13	17	F	None	None	Yes	Yes	Yes	4.6	FC
14	25	M	None	None	Yes	Yes	Yes	5.5	FC
15	20	F	Hypothyroidism	Levaxin	No	No	Yes	3	FC
16	25	M	None	None	No	Yes	No	13	FC
17	45	F	Hypothyroidism	Levaxin	No	Yes	Yes	9.2	FC
18	46	M	None	None	Yes	No	No	6.3	FC
19	19	F	None	None	No	No	Yes	6	FC
20	35	F	None	None	Yes	Yes	Yes	8	FC
21	35	M	None	None	No	No	Yes	6	FC/IF

F: Female, M: Male

SSRI: Selective serotonin reuptake inhibitor, 6-MP: Mercaptopurine, SNRI: Serotonin and noradrenaline reuptake inhibitor, GAS: Group A streptococcus, FC: Flow cytometry, IF: Immunofluorescence, PASI: Psoriasis Area and Severity Index.

a Positive GAS infection at onset, 8 months prior to inclusion.

### Reagents and resources

Information on the reagents, resources and instruments used in the study is provided in Table S1.

### Haematoxylin and eosin staining of GP skin biopsies

Stored biopsies were sectioned (7 μm) at the Biomedicum histological core facility (Histocore, Karolinska Institutet, Stockholm, Sweden) using Cryostar NX70. The slides were fixed in 4% formaldehyde for 5 min and then stained with haematoxylin and eosin following a standard protocol. First, they were submerged in MayersHTX for 5 min and then washed thoroughly with water. Afterwards, the slides were submerged in 0.5% eosin solution for 15 s, followed by serial washes with 70%, 95% and 99% ethanol. Finally, the samples were mounted on DPX mounting media and were visualised in a Leica DM4000B microscope.

### Immunofluorescence

Sectioned biopsies (7 μm) were fixed with pre-cooled acetone at room temperature (RT) for 5 min. Unspecific signal was blocked in a sequential manner with Image-iT™ FX Signal Enhancer, Background Buster and 10% heat-inactivated foetal calf serum (FCS) in Phosphate buffer saline (PBS) supplemented with 0.1% saponin for 30 min, and in between each blocking step the tissues were washed with PBS supplemented with 0.1% saponin. The sections were incubated with rabbit anti-LL-37, rat anti-HLA-DR and mouse anti-HNE at the dilutions mentioned in Table S2 overnight at 4 °C. Skin tissues were further blocked with 1% goat serum in PBS supplemented with 0.1% saponin for 30 min to block non-specific binding. Following this, the sections were stained with secondary antibodies: anti-rabbit IgG-Alexa Fluor 488, anti-rat IgG-Alexa Fluor 546 and anti-mouse IgG-Alexa Fluor 647 for 40 min at RT in the dark and then washed 3 times with PBS supplemented with 0.1% saponin (Table S2). The sections were mounted on slides with Prolong Diamond Antifade DAPI mounting media to stain the nucleus and imaged with a Nikon A1R confocal microscope.

### Cell isolation from skin

Punch biopsies from lesional skin were collected in RPMI 1640 medium supplemented with 10% FCS and were processed within 1 h of collection. Enzymatic digestion was performed in RPMI 1640 medium containing 0.25 mg/ml collagenase II and 0.25 mg/ml DNase I at 37 °C for 25 min under magnetic stirring. Cell suspension was then filtered through a 100 μm cell strainer and rinsed with RPMI 1640 medium supplemented with 10% FCS, followed by centrifugation at 1500 rpm for 5 min, removal of the supernatant and cell staining for flow cytometry.

### Cell staining and flow cytometry

For flow cytometry analysis, 200 μl of whole blood from healthy controls (n = 29) or patients with GP (n = 21) and matched lesional cell suspensions from 10 patients with GP were stained at room temperature with fluorescently labelled antibodies for cell profiling (Table S3) or cell sorting (Table S4). In brief, plasma from whole blood and medium from skin suspension were removed after centrifugation (1500 rpm for 3 min at RT). The isolated cells were washed with FACS buffer (PBS, 5% FCS, 0.05 mM EDTA), followed by a 10 min incubation with a cocktail containing Fc-block, fluorescently labelled antibodies for extracellular staining and LIVE/DEAD staining dye. Then, the cells were washed twice with FACS buffer and fixed with BD Cytofix/Cytoperm™ for 10 min. Subsequently, the cells were washed twice with 1x Perm/wash solution and incubated for 10 min with an antibody cocktail for intracellular staining. This was followed by two washes with 1x Perm/wash solution and final resuspension of the cells in FACS buffer. The samples were acquired immediately on a BD LSR Fortessa™.

In the case of staining for cell sorting, the skin lysates were washed twice with FACS buffer after extracellular staining, followed by immediate sorting on a SONY cell sorter MA900. Blood samples required lysis of red blood cells (RBC) before sorting. This was achieved through incubation of the stained cells with 1x RBC buffer at room temperature for 10 min, followed by washes with FACS buffer and resuspension in FACS buffer.

Phenotyping of the *in vitro* stimulated cells was performed immediately after the indicated time points. Briefly, the cells were washed twice with FACS buffer and incubated for 10 min at room temperature with a cocktail containing Fc-block, LIVE/DEAD stain and fluorescently labelled antibodies for extracellular staining (Tables S5 and S6). Then, the cells were washed twice with FACS buffer and fixed with BD Cytofix/Cytoperm™ for 10 min. Subsequently, the cells were washed twice with 1x Perm/wash solution and resuspended in FACS buffer. The samples were acquired immediately either on a BD FACSymphony equipped with five lasers (WBC stimulation) or on a Cytek Aurora cytometer equipped with five lasers (co-culture of CD4^+^ T cells with neutrophils).

### Flow cytometry data analysis

Flow cytometry data analysis was performed using FlowJo v10. A compensation matrix was generated using AutoSpill,^31^ optimised and applied to the fcs files. Following exclusion of doublets and dead cells, lymphocytes were defined as CD45^+^Lin(CD3/CD19/CD56)^+^, neutrophils as CD45^+^Lin^−^CD15^+^CD66b^+^CD16^bright/dim^CD14^−^CD193^−^, eosinophils as CD45^+^Lin^−^CD15^+^CD66b^+^CD193^+^CD16^−^ and monocytes/dendritic cells/macrophages collectively as CD45^+^Lin^−^CD15^−^HLA-DR^+^CD14^+/−^CD16^+/−^. The gating strategies for each experiment are included in the corresponding Supplementary Figures. Dimensionality reduction was performed with the uniform manifold approximation and projection (UMAP) FlowJo plugin v3.1. Live total CD45^+^ cells or live neutrophils from all included donors and conditions were downsampled for comparability (FlowJo Downsample plugin v3.3), barcoded, and concatenated. FlowJo Phenograph v3 was used for unsupervised clustering of neutrophils from blood and matched skin samples, or from the stimulated neutrophils from healthy donors, with the optimal k-nearest neighbours (KNN) implemented automatically. The HyperFinder tool in FlowJo was used to identify the gating strategy for Phenograph cluster 13 in the stimulated neutrophils from healthy donors (n = 3, initial cohort) and apply it to the UMAP generated from the samples of the validation cohort (n = 8).

### Cell sorting and single-cell RNA-sequencing (scRNA-seq) library preparation

Cell populations from matched blood and skin lesional samples (n = 4) were processed for sorting as described above. 30,000 live cells (20,000 neutrophils, 5000 CD3^+^ T cells and 5000 other cells) from each blood sample and around 1000 cells from each skin sample were sorted and immediately processed for generation of Gel Bead Emulsions (GEMs) in the Chromium controller, followed by reverse transcription (GEM-RT), cDNA amplification and library construction according to the Chromium™ Next GEM Single Cell 3′ reagent kit v3.1 user guide (10x Genomics). Quality controls for cDNA amplification and final barcoded libraries were performed using High Sensitivity D5000 Agilent Tapestation to assess the quantity and fragment size. 2.5 nM from each library (10 nM per lane) was loaded on Illumina NextSeq 2000 P3 50 (88 cycles total => R1 28bp, R2 52, bp I1 8bp).

### scRNA-seq data processing

#### Data alignment and normalisation

The fastQ files generated after sequencing of the samples were aligned and quantified using the module count from the Cell Ranger Software (version 5.0.1).^32^ *Homo sapiens* reference genome version GRCh38.96 downloaded from Ensembl was used as a reference. Genes annotated as protein coding or lncRNA were selected for the alignment. The count matrices of blood and skin tissue samples were then separately merged using the module aggr from the Cell Ranger Software. Further, all the downstream processing of the count matrix was performed using the R package Seurat (version 3.1.5).^33^ Quality control of the data was done by checking the total number of genes detected per cell and mitochondrial gene percentage. Genes detected in fewer than three cells, cells with detected <200 or >2500 genes and cells having >10% mitochondrial genes were removed. The count matrix was scaled down using ScaleData() function and variations arising from cell cycle scoring were regressed out. The data was normalised using NormalizeData() function with default settings.

#### Cell clustering and cluster annotation

RunPCA() function was used to generate principal components. An elbow plot was created to find the optimal number of PCs for cell clustering. The number of PCs was chosen by taking the point where the percentage of change in variation between two consecutive PCs was <0.1%. Further, FindNeighbors() and FindClusters() were used in default settings to compute shared nearest neighbour (SNN) graph and clusters of cells by SNN modularity optimisation-based clustering algorithm. Finally, RunUMAP() function was used for dimensionality reduction and identify clusters of cells. Marker genes specific to each cluster were identified using the FindAllMarkers (min.pct = 0.25, logfc.threshold = 0.25) function. The top 10 markers of each cluster were derived by ranking based on avg_log2FC, and a heatmap using DoHeatmap() was plotted to visualise the cluster differentiation. Cell clusters were annotated by checking the expression of canonical cell type markers.

#### Differential expression and pathway enrichment analysis

The FindMarkers (min.pct = 0.1, min.diff.pct = 0.2, logfc.threshold = 0.2) function from the Seurat (version 3.1.5) package^34^ was used to identify differentially expressed genes in each cell cluster between tissues. Further, genes expressed in each cell cluster compared to other clusters were found by using the FindAllMarkers (min.pct = 0.25, logfc.threshold = 0.25) function. Genes with padj < 0.05 were considered as significantly expressed. Pathway enrichment analysis was performed using the enrichr module from the tool gseapy (version 0.10.5).^35^, ^36^, ^37^ KEGG (Kyoto Encyclopedia of Genes and Genomes) pathway gene sets were used as a reference. The R package ggplot2 (version 3.3.5) was used to generate bubble and dot plots.

#### Neutrophil functional and granularity analysis

Gene ontology (GO) gene sets were used as a reference to assess the granularity of the identified neutrophil sub-populations. GO terms, namely, azurophilic granule (GO:0042582), specific granule (GO:0042581), gelatinase granule (GO:0070820) and secretory vesicle (GO:0099503), were chosen to study the granularity. Expression of genes associated with the selected GO terms was derived from the normalised gene expression table of total neutrophil cells, and the results were visualised as dot plots generated using the R package ggplot2 (version 3.3.5).

#### Comparison with publicly available datasets

Neutrophil datasets of healthy individuals and patients with bacterial acute respiratory distress syndrome (ARDS) were obtained from Sinha et al.^38^ The RunHarmony() function from the R package harmony^39^ was used to integrate healthy, ARDS and psoriasis data, and the batch effect was further corrected during the process. The RunPCA() function was used to generate principal components. An elbow plot was created to find the optimum number of PCs for cell clustering. The number of PCs was chosen by taking the point where the percentage of change in variation between two consecutive PCs was <0.1%. Further, FindNeighbors() and FindClusters() were used in default settings to compute the SNN graph and clusters of cells by SNN modularity optimisation-based clustering algorithm. Finally, the RunUMAP() function was used for dimensionality reduction and identification of clusters of cells. Marker genes specific to each cluster were identified using the FindAllMarkers (min.pct = 0.25, logfc.threshold = 0.25) function. The top 10 markers of each cluster were derived by ranking based on avg_log2FC, and a heatmap using DoHeatmap() was plotted to visualise the cluster differentiation.

#### Interactome analysis

scRNA-seq data from skin and blood of patients with GP were used to analyse interactions between neutrophils and other cells. Differential cell–cell interaction networks were constructed using CellPhoneDB, a publicly available repository of curated receptors, ligands and their interactions, to investigate communication or signalling interactions between cells in single-cell experiments.^34^^,^^40^ The total number of interactions and interaction strengths were calculated and plotted.

#### Cytokine and chemokine expression

Cytokine- and chemokine-related genes were selected and plotted using Seurat::DotPlot() function. The Seurat::DotPlot() uses predefined scales that display the average expression values on the colour scale and the percentage of cells within the group expressing the feature on the size scale. As the percentage of cells expressing the feature increases, the size of the dots on the plot increases accordingly.

#### Single-cell pseudotime and trajectory analysis

Using Slingshot,^41^ cluster Neu 0 (exclusively expressing precursor marker *CSF3R)* was set as the starting cluster, and two lineages of neutrophils were predicted using the default settings. Single-cell trajectory and pseudotime of neutrophils were visualised on the UMAP.

### Preparation of GAS for stimulation of white blood cells (WBC)

The bacterial cultures were inoculated from a single colony of *Streptococcus pyogenes* (strain 2006 from INFECT biobank)^42^ on Todd Hewitt broth with 1.5% yeast extract and incubated overnight at 37 °C. The bacteria were collected through centrifugation at 1600×*g* for 10 min. The supernatant was collected and filtered through a 0.2 μm filter. The bacterial pellet was washed once with PBS, and after centrifugation at 5000×*g* for 3 min at 4 °C the pellet was resuspended in 500 μl of CellFix, followed by 60 s vortex, 90 s incubation, 30 s vortex and addition of 500 μl PBS. The bacteria were then centrifuged at 5000×*g* for 3 min at 4 °C and resuspended in PBS. This step was repeated three times, and the bacteria were then resuspended in RPMI medium and used immediately for cell stimulation as described below.

### WBC stimulation

WBC were collected from 4 ml of EDTA blood from 11 healthy donors (n = 3 initial cohort, n = 8 validation cohort) after HetaSep™ depletion of RBC, according to manufacturer's instructions. The WBC were resuspended in RPMI 1640 medium supplemented with 10% FCS and 2 mM l-glutamine, followed by plating of 190 μl cells-medium per condition (corresponding to the WBC cells of 700 μl blood) in a well of a 96-well U-bottom plate and addition of 10 μl of one of the following: fixed GAS bacteria (final MOI (multiplicity of infection) 10 or 100), GAS culture supernatant, LPS (final concentration 40 μg/ml) or medium (unstimulated cells) for 4 h. After 4 h, the cells were washed with FACS buffer and stained with a cocktail containing Fc-block, LIVE/DEAD and fluorescently labelled antibodies (Table S5) as described above. The samples were acquired immediately on a BD FACSymphony.

### Co-culture of CD4^+^ T cells with neutrophils and assessment of T cell proliferation

Whole blood from healthy donors (n = 5) was processed with HetaSep™ for RBC depletion as described above. WBC were resuspended in RPMI 1640 medium supplemented with 10% FCS and 2 mM l-glutamine and incubated either with GAS bacteria (final MOI 3) or without for 2 h at 37 °C. Τhe stimulated and unstimulated leukocytes were washed twice in PBS containing 2% FBS and 1 mM EDTA. Subsequently, neutrophils and CD4^+^ T cells were purified by negative selection using the EasySep™ human neutrophil and EasySep™ human CD4^+^ T cell isolation kits, respectively, according to the manufacturer's instructions. Afterwards, the enriched CD4^+^ T cells were stained using the CellTrace™ CFSE as recommended. In parallel, the neutrophils were incubated in the presence or absence of HLA-DR, DP, DQ blocking antibody (20 μg/ml) for 1 h at 37 °C. CFSE-labelled enriched CD4^+^ T cells were seeded in each well of a 96-well U-bottom plate containing the enriched neutrophils (T cell:neutrophil ratio = 3:2) and cultured for 3 days in the presence of HLA-DR, DP, DQ blocking antibody (10 μg/ml). Additionally, GAS-CD4^+^ T cells were stimulated with Dynabeads® human T-activator CD3/CD28 and human IL-2 recombinant protein and cultured for 3 days to serve as a positive control. Staining and flow cytometry analysis were performed on day 0 for baseline assessment and on day 3 for evaluation of proliferation. Briefly, the cells were stained with a cocktail containing Fc-block, LIVE/DEAD and fluorescently labelled antibodies (Table S6) as described above and acquired immediately on a Cytek Aurora cytometer.

### Statistics

GraphPad Prism version 9.3.1 and the R package ggplot2 (version 3.3.5) were used to conduct statistical analyses, where p < 0.05 or padj < 0.05 as indicated in the figure legends were considered significant. The two-tailed and non-parametric Mann–Whitney U test was used for two-group comparisons, the non-parametric Kruskal–Wallis test for three-group comparisons and the non-parametric Friedman test for matched data. In the co-culture assay, normality was assessed using the Shapiro–Wilk test, and comparisons were performed using repeated-measures one-way ANOVA with Geisser–Greenhouse correction, followed by Šidák's post-hoc test for multiple comparisons. Where indicated, the Z-score of median fluorescence intensity (MFI) or RNA expression was calculated as follows: Z = (x−μ)/σ, where x = raw score, μ = mean of sample distribution and σ = standard deviation.

### Ethics statement

All blood and tissue samples were collected according to the principles of the Declaration of Helsinki, and the study was approved by the institutional review board of the regional ethics committee in Stockholm (Dnr 2012/50-31/2, amendment Dnr 2017/1774-32 and Dnr 2016/1415-32).

### Role of funders

The funding sources had no role in the study design, data collection and interpretation, analysis, writing of this report, or in the decision to submit the paper for publication.

## Results

### Distinct neutrophil subpopulations in lesional GP skin and blood

**Fig. 1:**
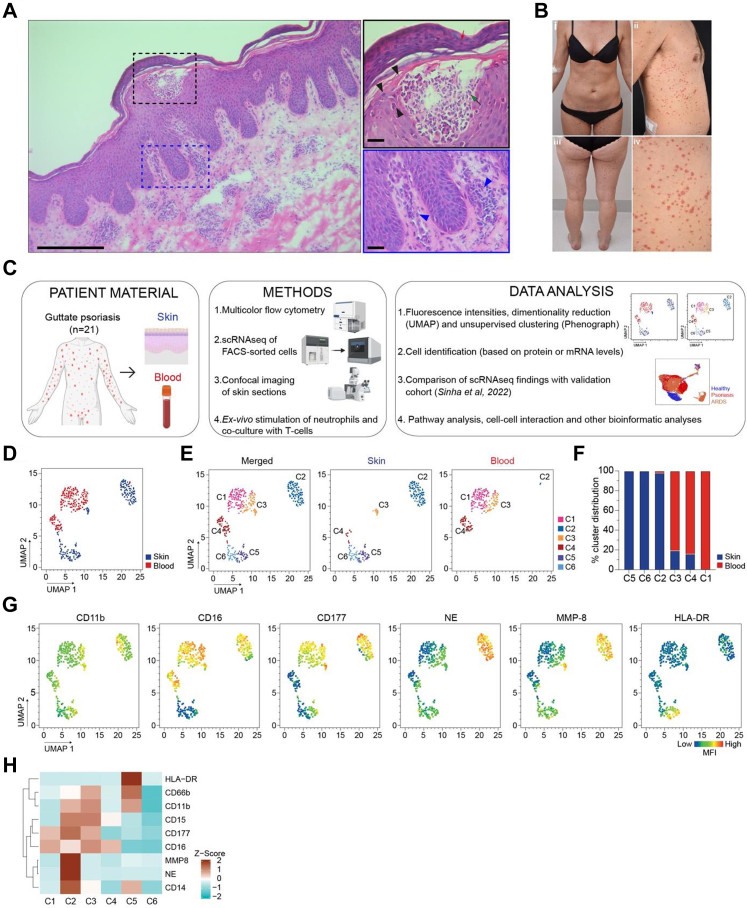
**Distinct neutrophil subpopulations in GP lesional skin and blood.** (A) Haematoxylin and eosin staining of lesional GP skin. Skin biopsy reveals infiltration of the epidermis by neutrophils (black box) and other immune cells (blue box and blue arrowheads). Neutrophil infiltration leads to the formation of neutrophilic vesicles (black arrowheads) and spongiform pustules of Kogoj (green arrow). Neutrophils were also present in the stratum corneum (red arrow). The scale bar indicates 250 μm; the zoomed-in panels indicate 50 μm. (B) Representative photos of patients with GP illustrating litttle, thin, red, scale-covered lesions on their arms, legs, or trunk. (C) Overview of the experimental and analytical workflow of the study performed on samples from GP lesional skin and blood. (D) Paired lesional skin (n = 6) and whole blood samples (n = 6) were processed and stained for multicolour flow cytometric analysis using the staining panel mentioned in Table S3. Live single neutrophils (CD45^+^/CD3^−^/CD19^−^/CD56^−^/CD14^−^/CD193^−^/CD15^+^/CD66b^+^) were gated, and dimensionality reduction was done to prepare a uniform manifold approximation and projection (UMAP) of total neutrophils (6 patients, 831 neutrophils/sample). (E) Distribution of the 6 Phenograph clusters overlaid on the UMAP space. The legend on the right indicates the colour of each Phenograph cluster. (F) Relative frequency of the Phenograph clusters in skin and blood. (G) Median fluorescence intensity (MFI) of selected neutrophil markers in the UMAP displayed in D. The colour key from blue to red indicates low to high expression levels. (H) Heatmap of the normalised expression (Z-score) of extracellular and intracellular markers in each neutrophil cluster. NE: neutrophil elastase, MMP-8: matrix metalloprotease-8, neu: neutrophils.

Neutrophils are scarce in normal skin but recruited in high numbers during inflammation.^43^^,^^44^ In psoriatic lesions, neutrophils infiltrate the dermis and epidermis and can form Kogoj or Munro's microabscesses (Fig. 1A). Given that their migration into the epidermis is an early sign of psoriatic inflammation in skin, we explored potential differences in the phenotype of neutrophils in patients with GP (Fig. 1B–Table 1). For this, paired lesional skin and blood samples were collected from the same individuals and processed as described in Fig. 1C and the Methods section. Multicolour flow cytometry analysis and dimensionality reduction with UMAP of 30,480 single live CD45^+^ cells from matched skin (n = 15,240 cells) and blood (n = 15,240 cells) from six patients with GP (Table 1, Table S3) revealed several differences in the frequency of the major immune cell types between skin and blood (Figures S1A–E). Focussing on neutrophils (CD45^+^Lin^−^CD15^+^CD66b^+^CD16^bright/dim^CD14^−^CD193^−^) (Figure S1C and S1F), subsequent UMAP analysis showed a clear difference in neutrophil phenotype between skin and blood (Fig. 1D), while unsupervised clustering with the Phenograph algorithm identified six distinct neutrophil clusters (Fig. 1E), with three of them being enriched in skin (C2, C5, and C6) and the others (C1, C3, and C4) mostly found in blood (Fig. 1F). This separation was based on differential expression of several surface markers (e.g., CD11b, CD16, CD177, and HLA-DR) and intracellular levels of neutrophil elastase (NE) and matrix metalloproteinase 8 (MMP-8) (Fig. 1G and H). Cluster C2, which represented 50% of the skin neutrophils, displayed higher expression of neutrophil activation and migration markers, NE, MMP-8 and CD177 (Fig. 1F–H). A subcluster in skin that exclusively expressed HLA-DR (C5, Fig. 1G and H) was one of the most notable differences between skin and blood neutrophils and presumably represented a sub-population of antigen-presenting neutrophils. Furthermore, the activation markers CD66b and CD11b were highly expressed on neutrophils of cluster C5, with CD11b also known to play an important role in antigen presentation^45^ (Fig. 1G and H). This data highlights the heterogeneous phenotype of neutrophils in the skin and blood of patients with GP, with skin neutrophils exhibiting an activated phenotype, indicating that they respond to signals from the site of inflammation.

### Enrichment of antigen-presenting neutrophil subpopulations in GP

**Fig. 2:**
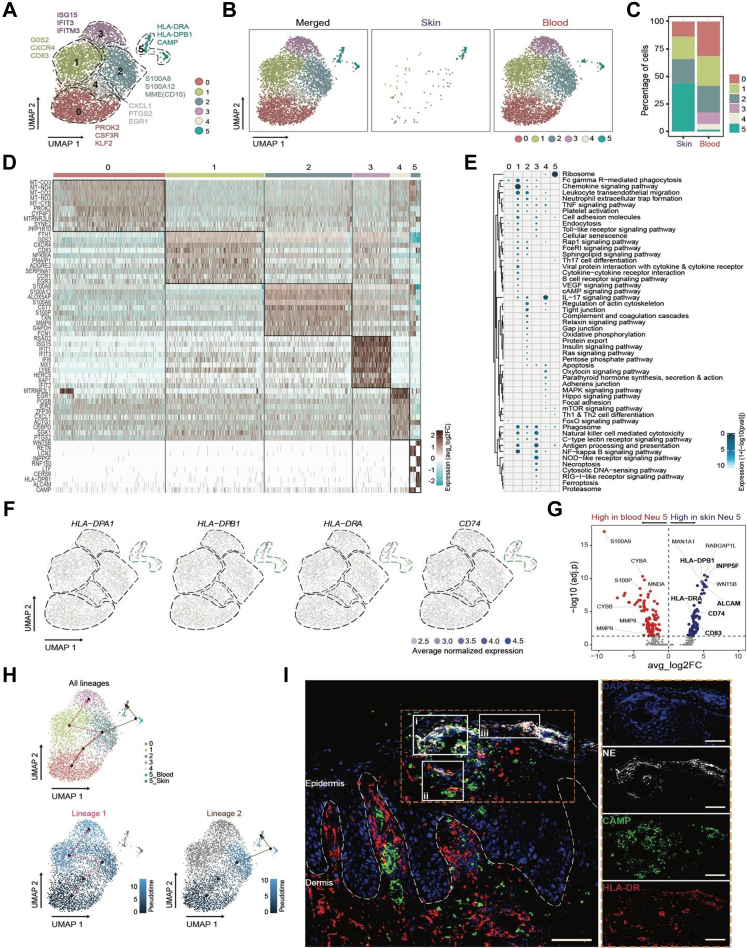
**Enrichment of antigen-presenting neutrophils in GP skin.** (A) Neutrophils were FACS sorted from matched blood and lesional skin samples (n = 4) and processed for scRNA-seq as described in the method section. UMAP visualisation of the six neutrophil subpopulations/clusters (0–5) from four patients with GP and representative marker genes for each neutrophil cluster, as determined by scRNA-seq. (B) UMAP visualisation of the distribution of the 6 neutrophil clusters in skin and blood. (C) Relative proportion of each neutrophil cluster in skin and blood. (D) Heatmap showing the top 10 genes expressed in each neutrophil cluster based on avg_log2FC (average log2 fold change). The colour key from turquoise to brown indicates low to high expression levels. (E) KEGG (Kyoto Encyclopedia of Genes and Genomes) pathway analysis with enriched gene ontology terms for each cluster. The colour key from light to dark blue and the dot size from small to big indicate low to high expression levels. (F) UMAP feature plots of antigen-presenting state-defining markers. (G) Volcano plot showing differential gene expression for neutrophils in cluster 5 from skin, compared to blood. (H) Pseudotime trajectory analysis (Slingshot) predicting the phenotypic progression from Neu 0 towards the other subpopulations. (I) Immunofluorescence protein staining of skin section for NE (white), HLA-DR (red) and LL-37 (green). Individual stainings are shown on the right inside the orange dotted box, while white boxes (i, ii, and iii) highlight areas of co-localisation. The dashed line indicates the border between the dermis and epidermis. The scale bar indicates 100 μm; the zoomed-in panels indicate 50 μm. KEGG: Kyoto Encyclopedia of Genes and Genomes, NE: neutrophil elastase, scRNA-seq: single-cell RNA-sequencing, UMAP: uniform manifold approximation and projection.

Next, we performed scRNA-seq on neutrophils sorted from matched skin and blood samples from four patients with GP (Figure S2A, Table S4). Transcriptomic analysis showed that neutrophils could be subclustered into six subpopulations (Fig. 2A) that differed in proportions between skin and blood (Fig. 2B and C) across patients (Figure S2B). The six subpopulations had distinct gene signatures (Fig. 2D–Table S7), expressed different levels of cytokine/chemokine receptors, produced different levels of cytokines/chemokines (Figure S2C) and had different functions (Fig. 2E, Figures S2D and E, Tables S8 and S9).

Cells in cluster 0 (Neu 0) were found to exclusively express *CSF3R* (Granulocyte colony-stimulating factor receptor), usually expressed by neutrophil “precursors” that respond to G-CSF stimulation, possibly initiating neutrophil mobilisation and differentiation into mature neutrophils in response to GAS infection (Fig. 2A–Table S7). Compared to other clusters, neutrophils in cluster 1 (Neu 1) exhibited an “aged” phenotype characterised by high *CXCR4* and low *CD62L* (*SELL*) expression, indicative of their role in resolution of inflammation by leaving circulation and trafficking into inflamed tissue (skin) (Fig. 2A and D, Figure S2C, Table S7). Genes encoding several S100 family proteins (*S100A12, S100A8, S100A6, S100A4*), *TXN* (thioredoxin) and *VIM* (vimentin) were highly expressed by cluster 2 cells (Neu 2, Fig. 2D–Table S7), commonly expressed in immature neutrophils, which are mobilised during infection. Clusters 3 and 4 appeared to have a mature phenotype based on high *MME* (Membrane Metalloendopeptidase), *CXCR4* and *FCGR3B* (CD16b) expression (Table S7). While cluster 3 (Neu 3) represented a distinct cellular subset with high levels of interferon (IFN)-inducible genes, including *ISG15, IFIT3,* and *IFITM3* (Fig. 2A and D, Table S7), often associated with pathogen clearance, cluster 4 neutrophils (Neu 4) had a less distinct phenotype, as none of the top genes expressed were unique for this cluster (Fig. 2D–Table S7). Functionally, neutrophils in cluster 0 expressed the suppressor of cytokine signalling 3 (*SOCS3*) (Figure S2C), a negative regulator of proliferative signals from G-CSFR,^46^ whereas Neu 1 were enriched in genes involved in the phagosomal pathway (Fig. 2E–Table S8). Neu 3 were specifically linked to the signalling pathways NOD-like receptor, cytosolic DNA-sensing, RIG-I-like, necroptosis and ferroptosis (Fig. 2E–Table S8), while Neu 4 showed high expression of molecules of the MAPK, Hippo and hormone signalling pathways, adherens junction and focal adhesion (Fig. 2E–Table S8). The lack of Neu 3 and Neu 4 in GP skin could be due to the overall low cell numbers in the skin.

Cluster 5 neutrophils (Neu 5) were enriched in skin (Fig. 2C) and showed high expression of major histocompatibility complex (MHC) class II molecules (*HLA-DPA1*, *HLA-DPB1*, *HLA-DRA*) and HLA-DR antigen-associated invariant chain (*CD74*) (Fig. 2F), indicating a possible role in antigen processing and presentation. This is in line with our flow cytometry data, where a subpopulation of skin neutrophils expressing HLA-DR (cluster C5, Fig. 1F–H) was detected. A comparison between Neu 5 from skin and blood showed that skin Neu 5 were characterised by the expression of factors associated with antigen processing and presentation (Fig. 2G, Figure S2F). We also detected higher expression of *CCL17*, *CCL22* and *ALCAM* (Fig. 2D and G, Figure S2C) in skin Neu 5, a gene signature usually associated with the professional antigen-presenting cell populations of dendritic cells (DCs). Together these molecules are also involved in trafficking of activated T lymphocytes to sites of inflammation, especially in skin.^47^^,^^48^ These neutrophils also had the highest expression levels of *CD24, CD66b* (CEACAM8) and of early granulopoiesis markers (*CAMP, S100A8/S100A9, LYZ*) and reduced levels of maturity-associated genes (*MME/CD10, FCGR3B/CD16, CXCR2*), consistent with an immature phenotype (Fig. 2D, Figure S2C, Table S7). Additionally, this subset in skin exclusively expressed genes encoding for *SOCS* 1, 2, 4, 6, and 7, a family of intracellular proteins that negatively regulate cytokine-mediated signalling (Figure S2C). Interestingly, Neu 5 in skin expressed high levels of *ANXA1* (Annexin A1), a gene that plays a role in glucocorticoid-mediated down-regulation of the early phase of the inflammatory response (Table S7), further highlighting a possible immunosuppressive function of this subset. Pseudotime trajectory analysis predicted two potential neutrophil lineages in GP (Fig. 2H), with blood Neu 5 likely being the precursor of skin Neu 5 (Fig. 2H, lineage 2).

The presence of the antigen-presenting neutrophil subset in lesional skin of patients with GP was confirmed by immunofluorescent staining for HLA-DR protein, co-localised with LL-37 (*CAMP*), and the standard neutrophil marker neutrophil elastase (NE) (Fig. 2I, Table S2). Additionally, neutrophils positive for either HLA-DR or LL-37 were detected.

Collectively, these findings demonstrate widespread neutrophil transcriptional heterogeneity in patients with GP, with accumulation of differentially activated neutrophil clusters in skin, suggesting involvement of these subpopulations in initiation or maintenance of inflammation in GP.

### Neutrophils acquire an antigen-presenting phenotype upon GAS bacteria stimulation

**Fig. 3:**
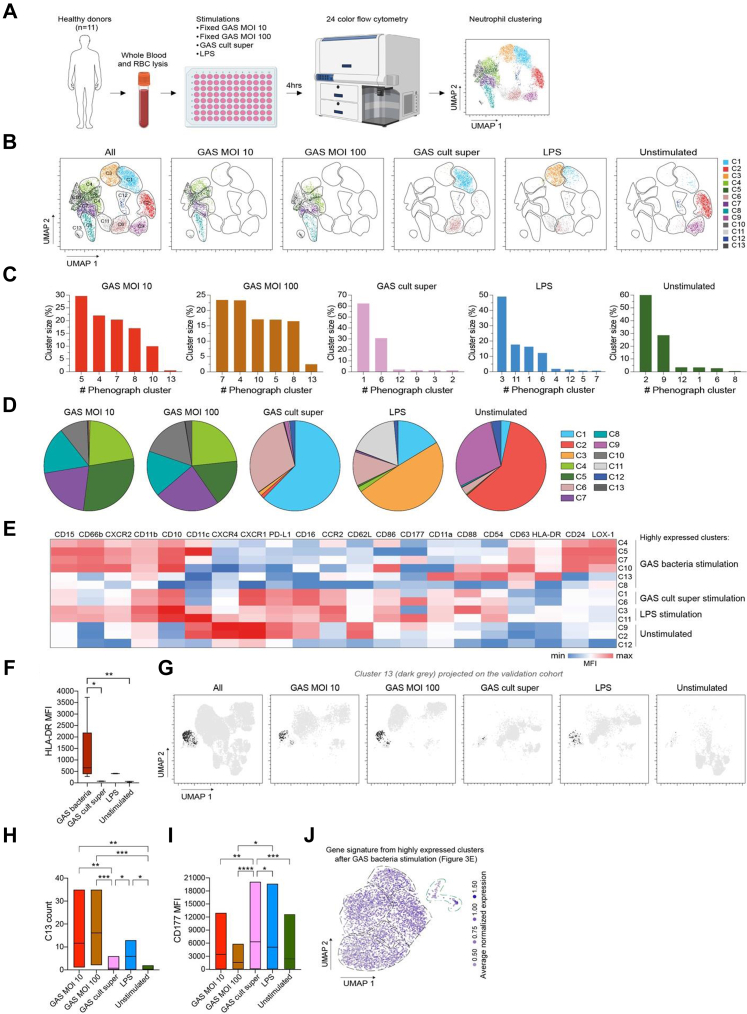
**Distinct neutrophil subpopulations following GAS stimulation.** (A) Experimental overview. GAS: Group A streptococcus, cult super: culture supernatant, LPS: Lipopolysaccharide. Cells isolated from whole blood samples of healthy controls (n = 3 initial cohort and n = 8 validation cohort) were stimulated with fixed GAS bacteria cultured overnight, culture supernatant, LPS or media control as shown in the figure. Following 4 h of stimulation, cells were stained with the flow cytometry panel presented in Table S5, acquired with a flow cytometer, and neutrophil data was analysed. (B) Distribution of the 13 Phenograph neutrophil clusters overlaid on the UMAP space after stimulation of white blood cells (n = 3) with either fixed GAS bacteria, GAS cult super, LPS or no stimulation. (C) Frequency of Phenograph clusters that are enriched after each condition. (D) Pie charts displaying the frequency of neutrophil Phenograph clusters after each condition. The legend on the right indicates the colour of each Phenograph cluster. (E) Heatmap of the normalised expression (Z-score) of median fluorescence intensity (MFI) of the markers in each neutrophil cluster. The colour key from blue to red indicates low to high expression levels. (F) HLA-DR MFI in the HLA-DR^+^ clusters upon GAS bacteria stimulations compared to HLA-DR MFI in the clusters enriched in the other conditions as indicated in E. (G) Projection of cluster 13 (C13) with the highest HLA-DR expression as shown in (E), on the UMAP space of the validation cohort (n = 8). (H) Total number of C13 cells across different conditions of the validation cohort, as shown in (G). (I) CD177 MFI in neutrophils from all donors (n = 11) following different stimulations. (J) UMAP visualisation of overlaying of average gene expression of GAS-induced “top expressed” proteins in neutrophils from our data in (E) on the scRNA-seq data of GP skin and blood. Statistical analysis in (F) was done using the non-parametric Kruskal–Wallis test, while in (H) and (I) the non-parametric Friedman test for matched data was used. ∗ indicates *p* < 0.05, ∗∗*p* < 0.01, ∗∗∗*p* < 0.001 and ∗∗∗∗*p* < 0.0001.

It has been long known that GAS throat infection can trigger and exacerbate GP,^49^ but the underlying mechanisms have remained largely unexplained. To determine if GAS infection in GP could be one of the driving factors for enrichment of this antigen-presenting subset of neutrophils, *ex vivo* stimulation of white blood cells isolated from blood samples of three healthy controls with fixed GAS bacteria was performed, followed by flow cytometry analysis (Fig. 3A, Table S5). Dimensionality reduction with UMAP and unsupervised clustering with Phenograph of live neutrophils (Figure S3A) resulted in 13 clusters of phenotypically different neutrophils (Fig. 3B). Clusters enriched after GAS bacteria stimulation exhibited a distinct activated phenotype (CD15^+^CD66b^+^CD24^+^CD62L^low^CD16^dim^) (Fig. 3C–E, Figures S3B–C), with significant upregulation of HLA-DR (Fig. 3E and F), and two of them (clusters 4 and 10) expressing CD86 at comparable levels (Fig. 3E, Figure S3B), indicative of their association with antigen presentation. Of note, cluster 13 expressed the highest levels of HLA-DR and activation markers CD11a and CD54 amongst all clusters and had high levels of CD11b, CD88 and the degranulation marker CD63, a phenotype indicative of reaction to bacteria (Fig. 3E). Projection of cluster 13 on the UMAP of a validation cohort (n = 8) showed a higher presence of these cells upon GAS stimulation as compared to other conditions (Fig. 3G–H, Figure S3D). A well-known inflammatory mediator with potent immunosuppressive activity, lectin-type oxidised LDL receptor-1 (LOX-1),^50^ undetectable in unstimulated neutrophils, was highly upregulated after stimulation with bacteria (Fig. 3E, Figure S3B). Neutrophil clusters with diverse phenotypes were seen in the LPS control and supernatant from the GAS bacterial culture; however, some clusters shared defining characteristics (Fig. 3B–E, Figure S3C). Of note, GAS bacteria stimulation resulted in CD177 downregulation on neutrophils (Fig. 3I), which might explain the decreased frequency of CD177^+^ neutrophils in patients with GP (Figure S3E). Together, this data indicates that peripheral blood neutrophils could alter phenotypic states in response to GAS bacteria, with enrichment of HLA-DR^+^ subsets. Mapping of this GAS bacteria-induced signature, i.e., genes coding for top expressed surface markers upon GAS stimulation as listed in Table S10 and Fig. 3E, onto our single-cell neutrophil dataset showed enrichment of this signature in both skin and blood Neu 5, but also expression of the signature in the other neutrophil clusters at various intensities (Fig. 3J, Figure S3E), indicating that GAS infection in GP could be one of the factors driving these activated neutrophils into the skin. Next, to confirm this observation, we projected a publicly available bulk RNA-seq dataset for host responses during symptomatic GAS pharyngitis^51^ (Table S10) and noticed enrichment of the gene signature in blood Neu 5 (Figure S3F) and presence in additional clusters in both skin and blood. In summary, GAS infection could be one of the factors driving neutrophil heterogeneity in GP and enrichment of the antigen-presenting neutrophils.

### Comparable neutrophil clusters in GP and bacterial ARDS

**Fig. 4:**
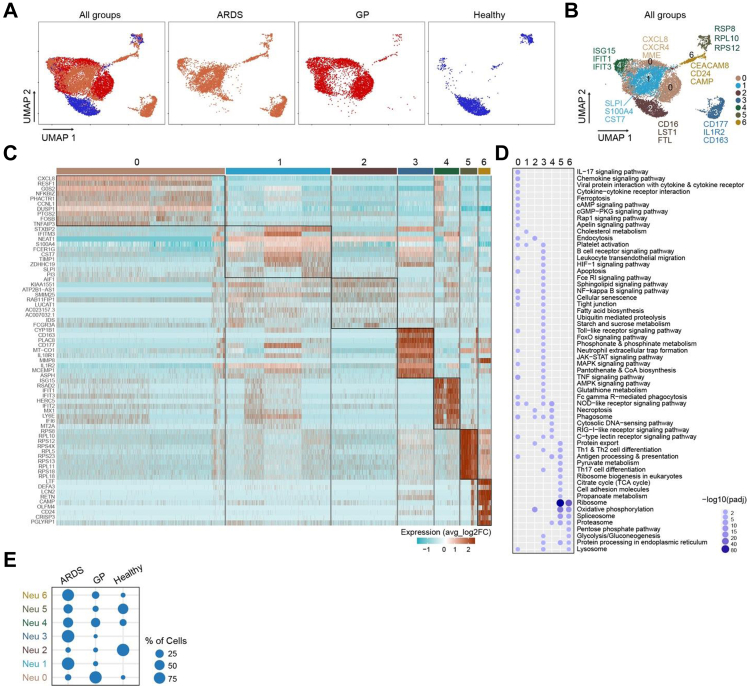
**Antigen-presenting neutrophils in GP and bacterial ARDS.** (A) Overall and individual UMAP visualisation of the transcriptome of blood neutrophils from patients with bacterial ARDS (n = 5), GP (n = 4) and healthy controls (n = 5). (B) UMAP visualisation of the seven identified neutrophil clusters as determined by analysis of scRNA-seq data. (C) Heatmap showing the top 10 genes expressed in each neutrophil cluster based on avg_log2FC (average log2 fold change). The colour key from turquoise to brown indicates low to high expression levels. (D) KEGG (Kyoto Encyclopedia of Genes and Genomes) pathway analysis with enriched gene ontology terms for each cluster. The colour key from light to dark purple and the dot size from small to big indicate low to high expression levels. (E) Proportion of each neutrophil cluster in ARDS, GP, and healthy controls.

Next, to investigate if distinct neutrophil endotypes were determinants of disease, we compared our transcriptomic data of blood neutrophils from patients with GP with published datasets from patients with bacterial ARDS, a neutrophil-dominant lung disease, and from healthy donors.^38^ UMAP analysis of the transcriptomic data from all three groups revealed that neutrophils in ARDS and GP clustered closer together compared to healthy controls (Fig. 4A). Neutrophil clustering showed seven separate clusters (Fig. 4B, Figure S4A) with distinct gene signatures (Fig. 4C–Table S11) and functions (Fig. 4D–Table S12) and differential distribution across healthy controls, GP, and bacterial ARDS (Fig. 4E). KEGG pathway analysis revealed that neutrophils in clusters 0, 4, and 5 expressed molecules of pathways involved in antigen processing and presentation (Fig. 4D), with clusters 0 and 5 showing the highest expression levels of *HLA-DRA*, *HLA-DPA1, HLA-DPB1*, *HLA-DQA1* and *CD83* (Table S11). Interestingly, blood neutrophils in GP exhibited significant expansion of cluster 0 as compared to ARDS and healthy controls, whereas clusters 4 and 5 were distributed across all three groups (Fig. 4E). However, none of the genes associated with antigen presentation was amongst the most highly expressed genes in any of the clusters (Table S11), suggesting that blood neutrophils do not exhibit such a pronounced antigen-presentation phenotype as compared to that of GP skin. Additionally, differential gene expression analysis (Figure S4B) and comparative granule analysis (Figure S4C) confirmed that the Neu 6 cluster resembled the previously identified Neu 5 population in GP. Thus, blood neutrophils in GP are associated with an antigen-presenting phenotype, but this is not exclusive to GP.

### Cellular crosstalk of neutrophils with other cell types likely contributes to the proinflammatory environment in GP

**Fig. 5:**
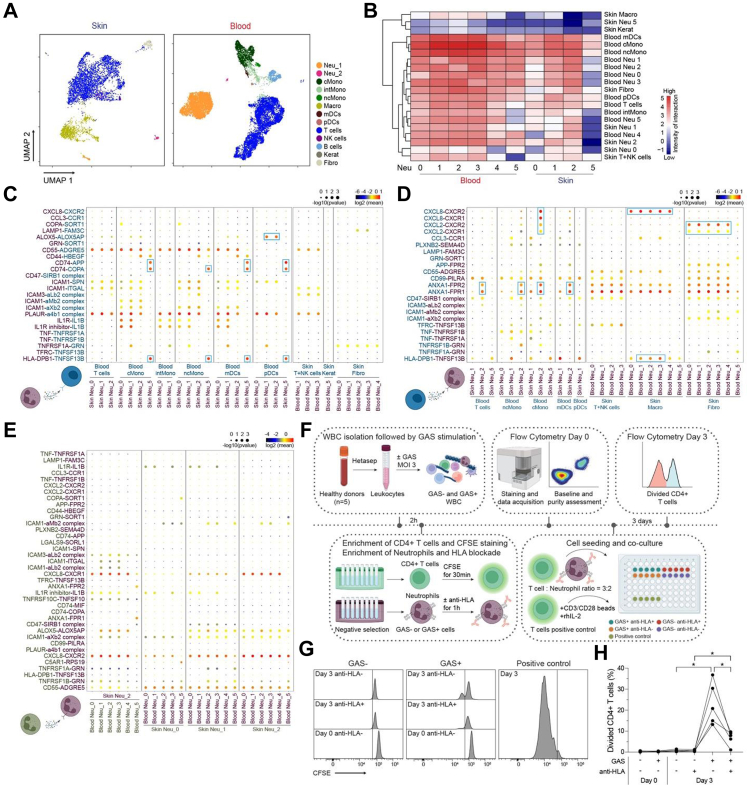
**Crosstalk between neutrophils and other cells in GP blood and lesional skin.** (A) Identification of cell populations in GP skin and blood obtained through unsupervised analysis of the scRNA-seq data and projection in the UMAP space. Neu 1 and Neu 2 were pooled together and subclustered as described in Fig. 2 (Neu 0–5) for the subsequent analyses. (B) Heatmap showing the number of significant interactions between neutrophil subpopulations and other cells in skin and blood as determined by applying the CellPhoneDB algorithm. The colour key from blue to red indicates low to high intensity of interaction. (C and D) Interaction strength (dot colour) and significance (dot size) of selected ligand-receptor pairs between neutrophil subpopulations and other cells. In (C), neutrophils produce the ligands (purple), which bind to receptors expressed by other cells (blue), while in (D) neutrophils express the receptors (purple) for the ligands secreted by other cells (blue). (E) Interaction strength (dot colour) and significance (dot size) of selected ligand-receptor pairs between neutrophil subpopulations in skin and blood. Neutrophils secreting the ligands are indicated in green, while neutrophils taking up the ligands are indicated in purple. (F) Schematic representation of CD4^+^ T cell-neutrophil co-culture assay. White blood cells were isolated from healthy blood donors (n = 5) and either left unstimulated or stimulated with overnight-grown fixed GAS bacteria at an MOI of three for 2 h. Post-stimulation, neutrophils and autologous CD4^+^ T cells were magnetically enriched. Neutrophils with or without anti-HLA antibody were co-cultured with CFSE-labelled CD4^+^ T cells for 3 days. CD4^+^ T cells cultured without neutrophils in the presence of anti-CD3/CD28 beads and human IL-2 recombinant protein (30 U/mL) served as a positive control for proliferation. (G) Representative flow cytometry histograms from one donor showing the frequency of divided CD4^+^ T cells as measured by decreased CFSE MFI of the live CD4^+^ T cells for the indicated conditions. (H) Plot showing percentage (%) of divided CD4^+^ T cells from five donors when cultured with autologous neutrophils under various conditions. Statistics in H were performed using repeated-measures one-way ANOVA with Geisser–Greenhouse correction, followed by Šidák's post-hoc test for multiple comparisons. ∗ indicates *p* < 0.05. Neu: neutrophils, cMono: classical Monocytes, intMono: intermediate Monocytes, ncMono: non-classical Monocytes, Macro: macrophages, mDCs: myeloid DCs, pDCs: plasmacytoid DCs, NK: natural killer cells, Kerat: keratinocytes, Fibro: fibroblasts. GAS: Group A streptococcus, HLA: Human leucocyte antigen, CFSE: Carboxyfluorescein succinimidyl ester.

Using a broad gating strategy for cell sorting from skin and blood (Figure S2A), subsets of lymphocytes, monocytes and DCs (blood only), macrophages (skin only), and structural cells (fibroblasts, keratinocytes; skin only) were analysed in addition to neutrophils (Fig. 5A, Figures S5A–E, Tables S13–S14). This allowed us to understand the interplay between the neutrophil subpopulations (Fig. 2A and B) and other cell types through investigation of cell–cell communication mediated by ligand-receptor complexes using CellPhoneDB.^40^ Various cellular interactions between the cell types were noted, with monocytes, DCs and T cells displaying the highest number of predicted interactions with all neutrophil subsets (Fig. 5B). The mature neutrophil subsets were found to have higher predicted interactions as compared to the immature subset (Neu 5), both in terms of number and strength of intercellular interactions (Fig. 5B–Table S15). Notably, an interaction exclusive to the immature neutrophil subset was between CD74 and the cell surface receptors APP and COPA on blood monocytes and DCs, as well as between HLA-DPB1 on skin Neu 5 and TNFSF13B on blood monocytes and DCs (Fig. 5C). Neutrophils in skin and blood were also sensitive to factors derived from other immune and non-immune cells (Fig. 5D). For example, skin fibroblasts produced CXCL2 and CXCL8 that bound to CXCR1 and CXCR2 receptors, likely attracting neutrophils to the site of inflammation. Amongst skin neutrophils, Neu 2 showed marked interaction through Annexin A1 (ANXA1-FPR1/2) with blood T and myeloid cells as well as through chemokine signalling with classical monocytes (Fig. 5D). Multiple interactions between the neutrophil clusters in skin and blood were also observed, but no cluster-specific interactions were observed (Fig. 5E). Thus, our findings suggest that neutrophils likely interact with other skin and circulating cells through a web of cell-to-cell interactions, possibly contributing to the proinflammatory environment and the attraction and retention of immune cells in psoriatic skin driving GP pathogenesis.

Next, we sought to functionally determine if GAS-stimulated neutrophils could influence T cell responses using a co-culture assay as described previously^16^^,^^52^ (Fig. 5F). For this, white blood cells isolated from whole blood of healthy individuals were pre-incubated with fixed GAS bacteria. Subsequently, the stimulated cells were magnetically enriched for neutrophils and CD4^+^ T cells. Neutrophils were cultured together with CFSE-labelled autologous CD4^+^ T cells for three days, and their ability to present antigens to T cells was assessed by measuring the proportion of CD4^+^ T cells that underwent division (CFSE dilution) (Fig. 5F). T cells showed no proliferation when co-cultured with unstimulated neutrophils, whereas significant proliferation occurred in the presence of anti-CD3/anti-CD28 beads and rhIL-2, as expected (Fig. 5G). GAS-stimulated neutrophils showed significantly higher antigen presentation capacity as evidenced by the high frequency of proliferating CD4^+^ T cells (Fig. 5G and H), with significantly reduced proliferation in the presence of a neutralising antibody against HLA-DR (Fig. 5G and H). No proliferation was seen when CD4^+^ T cells were cultured with HLA-DR-blocked neutrophils without antigen (Fig. 5H). This data clearly shows that the neutrophils that upregulate HLA-DR upon GAS stimulation possess the capacity to present antigens to T cells.

## Discussion

Tonsillar infection with GAS is a triggering factor for the onset of GP; however, the precise pathologic mechanism behind this association is still unresolved. In psoriasis, neutrophils are the foremost immune cells to infiltrate the skin before psoriatic lesion formation and play a critical role in the early phase of inflammation. Using high-dimensional, single-cell protein and RNA expression analyses, we have delineated neutrophil subsets in the skin and circulation of patients with GP. By extending our analysis to publicly available resources in combination with immunostaining of skin sections and *ex vivo* stimulation assays, we identified a subpopulation of activated neutrophils expressing HLA-DR that likely expands in response to GAS in lesional skin.

Neutrophils are amongst the first cells to be recruited from blood to the site of inflammation. The properties of neutrophils in psoriatic skin have been studied in various settings, often by flow cytometry.^29^ Recent advances in scRNA-seq have allowed the identification of immune cell subsets and broadened our understanding of many aspects of skin immunopathogenesis.^53^, ^54^, ^55^, ^56^ However, due to technical challenges in the workflow, neutrophils have often been under-represented or absent in these studies. Here we employed a multipronged approach to immunophenotype neutrophils, which revealed that neutrophils in the skin and blood of patients with GP are highly heterogeneous and span from immature to IFN-reactive and other mature subsets. During GP, the local inflammatory environment possibly influences their activation, maturation, and ultimately the function they serve within the context of the disease. While certain subsets of neutrophils (Neu 0, 2, and 5) may be involved in amplifying the inflammatory response, others seem to have an immunosuppressive phenotype promoting clearance and resolution of inflammation. Interestingly, the distribution of these subsets in skin and blood revealed that skin neutrophils displayed an activated phenotype, in contrast to circulating subsets. Of note, GAS has been detected at low levels in the blood of patients with GP, and it is likely that neutrophils in the blood sense and respond to cues from pathogen-associated molecular patterns (PAMPs).^57^ However, the majority of patients do not show persistence of GAS in skin, which indicates that during ongoing pharyngeal infection, GAS has the potential to drive cutaneous inflammation through a local response.^58^ This is through antigen-specific T cells that respond to GAS, get activated and acquire skin-homing receptors.^59^^,^^60^ Recent studies demonstrate that T cells can deliver signals to neutrophils, modulating their activation and function, with Th17 cells in particular known to drive recruitment of neutrophils to sites of infection through IL-17–induced chemokine production by keratinocytes and stromal cells.^61^ The neutrophil subpopulation that expanded specifically in GP skin was an *HLA-DR*^+^or MHC-II^+^subset with high expression of co-stimulatory molecules. Antigen-presenting function in neutrophils is in fact induced by interaction with activated T cells.^61^^,^^62^ Reciprocally, neutrophils can enhance T cell responses either directly or indirectly through adjacent myeloid cells.^63^ Indeed, circulating neutrophils from healthy donors upon stimulation with GAS were found to activate T cells in an HLA-DR-dependent manner. Our cell–cell interaction predictions further highlight this potential crosstalk between neutrophils, T cells and other antigen-presenting cells. The bi-directional interaction observed between neutrophils in skin and blood immune cells or neutrophils in blood with skin immune and stromal cells indicates potential modulation of each other's recruitment to inflamed tissue and function.

Induction of MHC-II and costimulatory molecules on neutrophils in the presence of antigen has been reported previously.^16^ Moreover, recent studies have described such functional plasticity and the ability of neutrophils to present antigens.^18^^,^^64^^,^^65^ Additionally, the emergence of such an HLA-DR^+^neutrophil population in circulation and local lesions of patients has been reported previously in patients with cutaneous leishmaniasis.^66^ However, we found exclusive enrichment of this population at the site of inflammation i.e., skin. Thus, these antigen-presenting neutrophils could be either a distinct immature subset that infiltrated the skin and got activated or a partially activated version of another subset, likely blood Neu 5. It is possible that the presence of antigens alone could induce neutrophil activation and gain of antigen-presenting functions, as was seen with our fixed bacteria stimulations.

Neutrophils are accepted to have multiple phenotypes; however, little is known with regard to the mechanisms that induce the appearance of one over another.^67^ We noted that the GAS-induced immature neutrophil population showed dual properties of antigen presentation with antimicrobial function. This indicates an alternative strategy employed by the immune system to control the bacterial reservoir, likely via production of antimicrobial peptides, such as cathelicidin. Such a unique neutrophil population that can participate in both innate and adaptive immune responses has been shown previously in mouse models.^68^ Additionally, the Neu 5 cluster showed the highest expression of Annexin A1 (*ANXA1)*, which is known to play a role in immunosuppression and resolution of inflammation through FPR2 signalling on T cells. However, upon GAS stimulation, circulating HLA-DR^+^ neutrophils (C13) lacked expression of the immunosuppressive marker LOX-1 and did not suppress T cell proliferation *in vitro*, highlighting the need for future studies to understand if the phenotype adapted by the HLA-DR^+^ neutrophil population is influenced by other factors. Therefore, HLA-DR^+^ neutrophils represent an immature phenotype that is partially activated, and their capability to activate T cells indicates a potential involvement in adaptive immunity, rendering them as candidates for immune profiling in clinical trials. Moreover, this population may serve as a biomarker for disease severity and immune dysregulation and could be used for monitoring therapeutic responses to immunomodulatory treatments. However, further studies are warranted to define their prognostic utility.

While our workflow provided robust and reproducible results concerning the alterations of neutrophil phenotypes in GP, it is too early to speculate what underlying mechanisms drive neutrophil state polarisation. Even though we used cells from freshly harvested skin, we cannot rule out the effect of enzymatic digestion, FACS sorting and scRNA-seq library preparation on neutrophils. Given that we detected proportionally few neutrophils in skin, it is also important to acknowledge these technical challenges in order to make reliable conclusions. Therefore, experimentally characterising possible functional differences between subsets is an important future goal. Our study is also limited by a lack of data on transcriptional states of neutrophils at steady state, due to their absence in healthy skin. Thus, comparison with circulating neutrophils from patients with bacterial ARDS and not with neutrophils from healthy skin, precludes assessment of whether the subsets are specific for GP skin.

Overall, this study adds to our understanding of neutrophil heterogeneity at disease sites, an area that has lagged behind compared to other immune cell subsets. By linking GAS infection with this antigen-presenting neutrophil subset, we suggest a broader interpretation of the fundamental biology of bacterial-associated inflammation, especially in GP. Future studies should determine whether HLA-DR^+^ neutrophils can serve as biomarkers not only in GP but also in other neutrophil-mediated psoriasis phenotypes.

## Contributors

Conceptualisation: AP, JL, ML. Methodology: AP, PA, ML. Investigation: AP, PA, MA, RSR, LPM, ML. Data analysis: AP, ATA, PA, IS, WZ, ML. Preparation of the figures: AP, ATA, PA, IS, WZ, ML. Patient recruitment and clinical information: JL. Resources: AP, JIH, MS, LE, ANT, UN, PB, JL, ML. Supervision: ML. Writing of the manuscript: AP, PA, PB, JL, ML. All authors reviewed and approved the final version of the manuscript. Flow cytometry data have been accessed and verified by AP, PA and ML. scRNAseq data have been accessed and verified by ATA, IS, WZ and ML. Immunofluorescence data have been accessed and verified by AP, RSR, LPM and ML.

## Data sharing statement

The scRNA-seq raw and processed data used in the paper are available in the NCBI Sequence Read Archive (SRA) with submission number SUB13682758. The codes used in the manuscript are available in the Github (https://github.com/neogilab/scRNA-Psoriasis) repository. Flow cytometry fcs files can be provided by the corresponding author ML upon reasonable request.

## Declaration of interests

LE has served as a paid speaker by Johnson & Johnson in a dermatology course. JL has served as a paid speaker and received funding for engagement in scientific boards and moderatorship for educational events for Abbvie, Johnson & Johnson, Leo Pharma, UCB Pharma, Lilly, Novartis and Sanofi. The other authors have no conflict of interest to declare.

## Appendix A Supplementary data

Supplementary Tables

Graphical Abstract

Caption for supplementary material
