# Supplementary material for: High-dimensional single-cell analyses reveal neutrophil heterogeneity in guttate psoriasis
Source: eBioMedicine. 2026 Feb 19;125:106172. doi: 10.1016/j.ebiom.2026.106172 (PMC12936745; doi:10.1016/j.ebiom.2026.106172)

Supplementary Figure 1

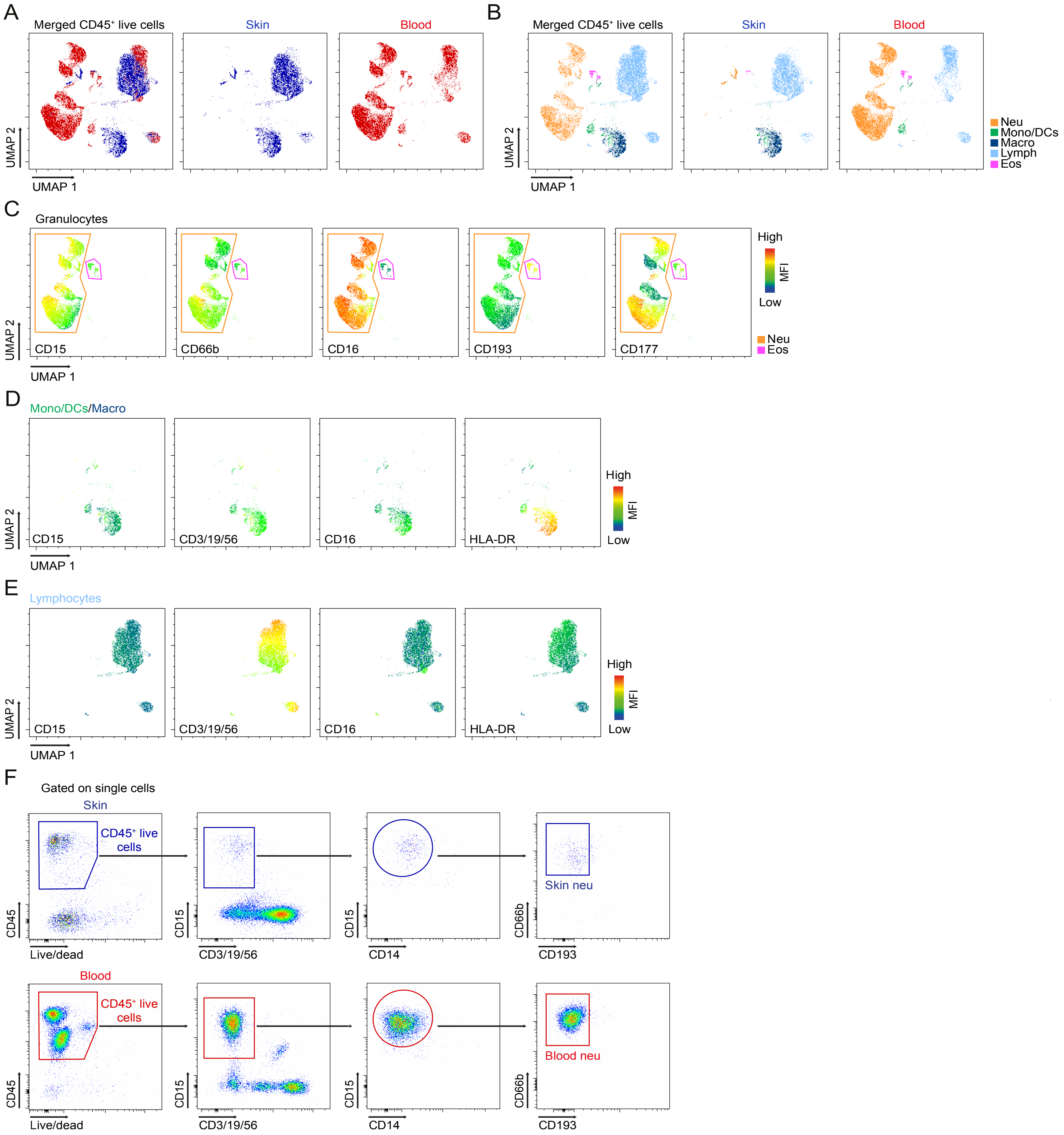

Supplementary Figure 2

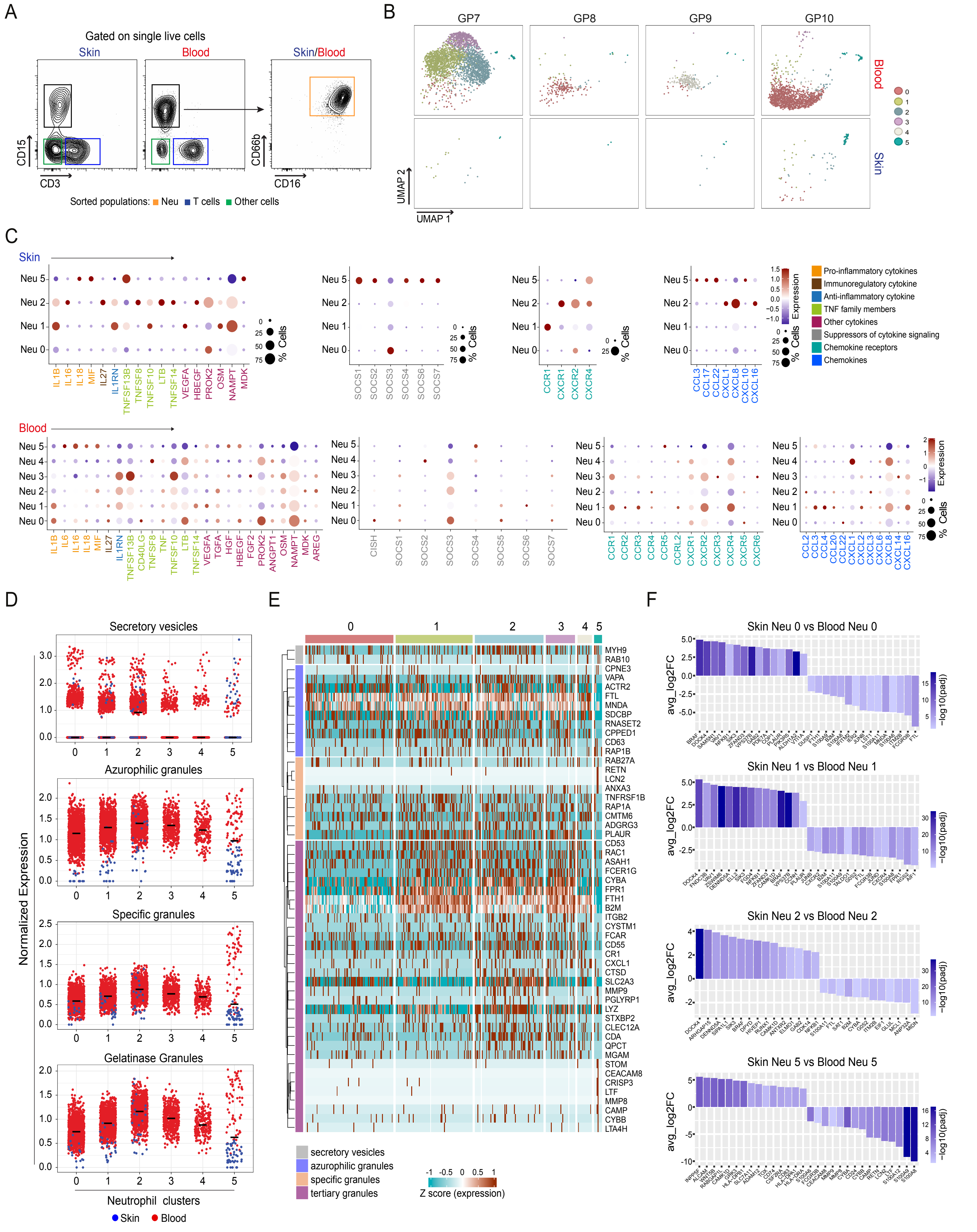

Supplementary Figure 3

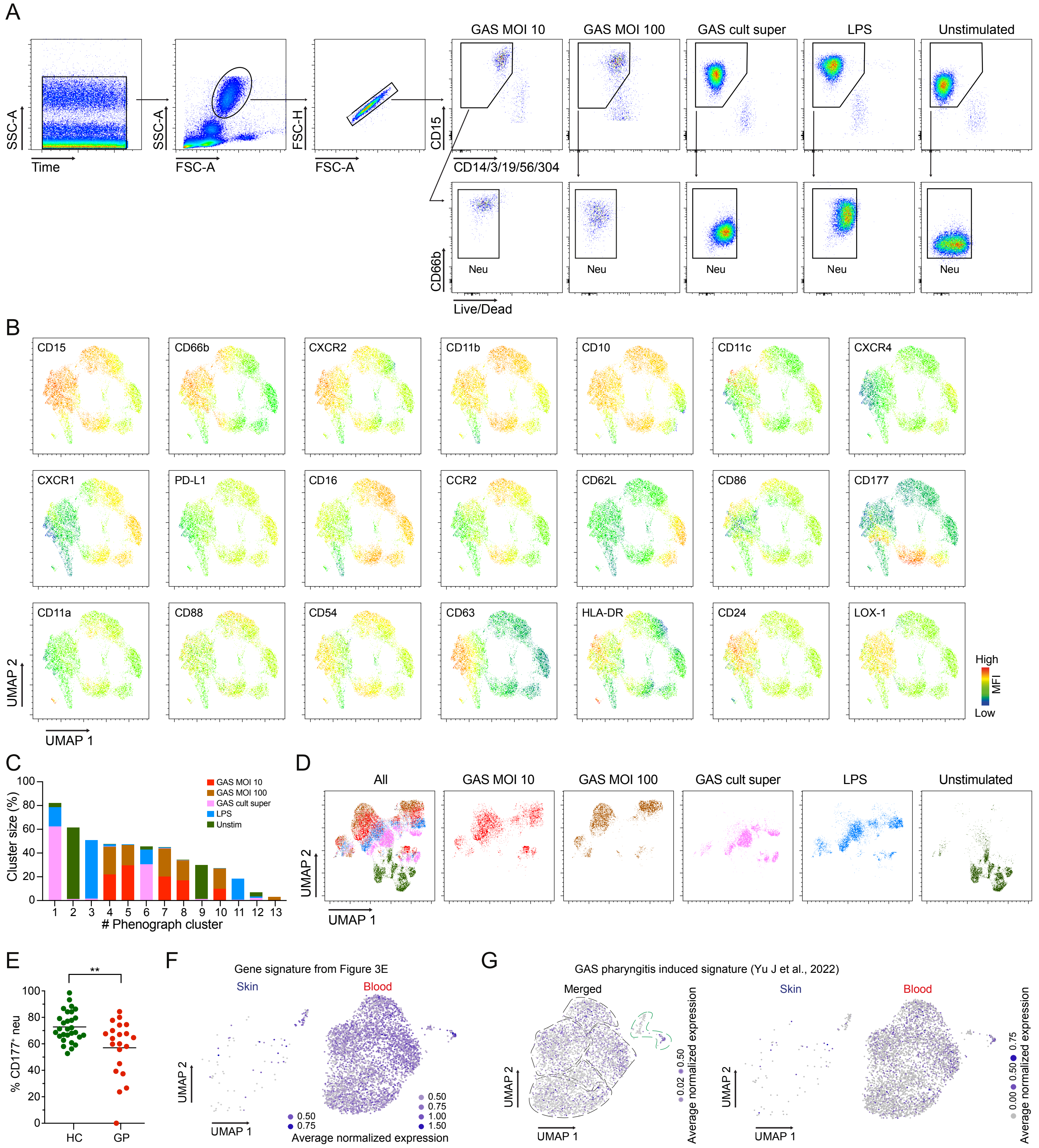

Supplementary Figure 4

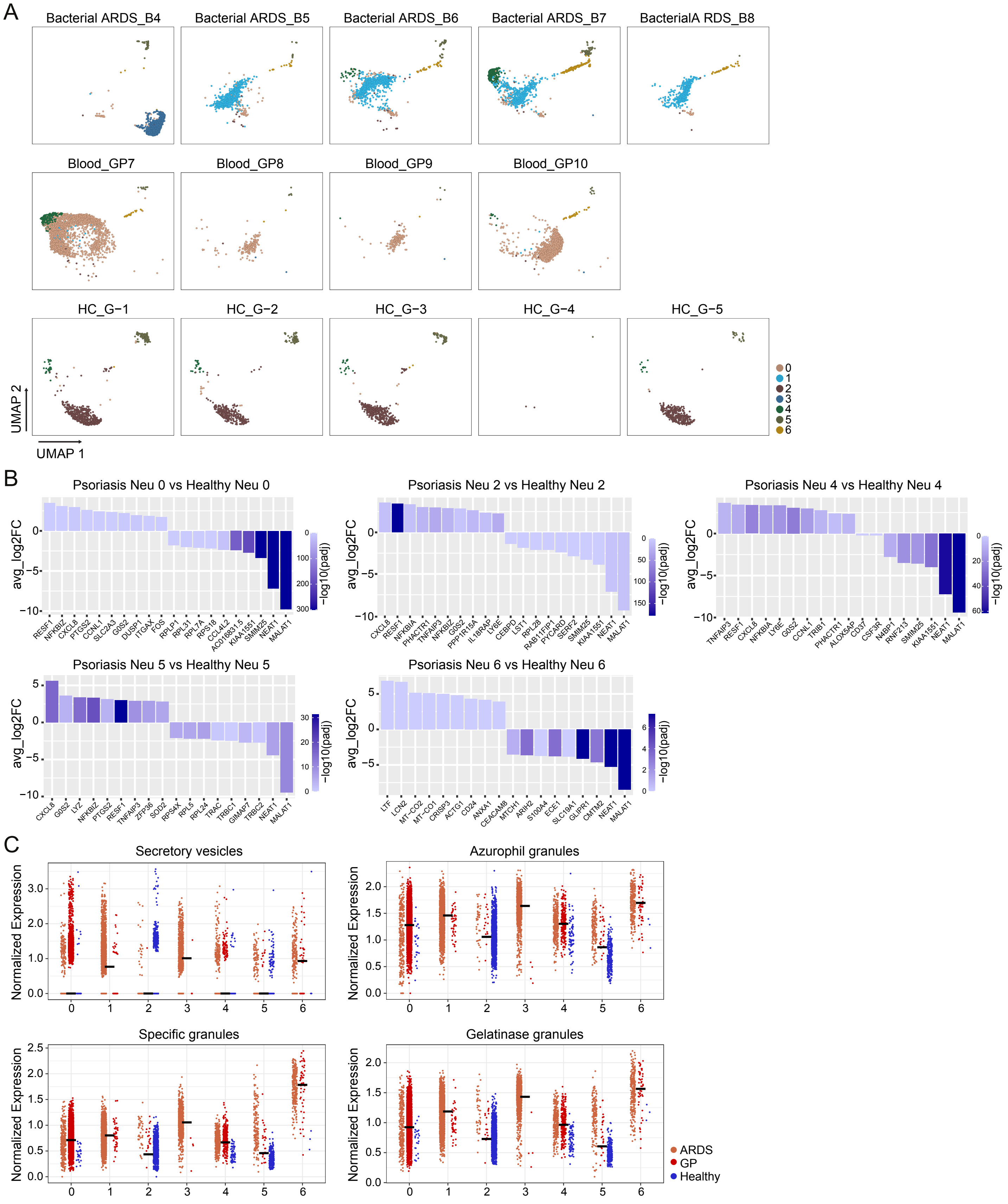

Supplementary Figure 5

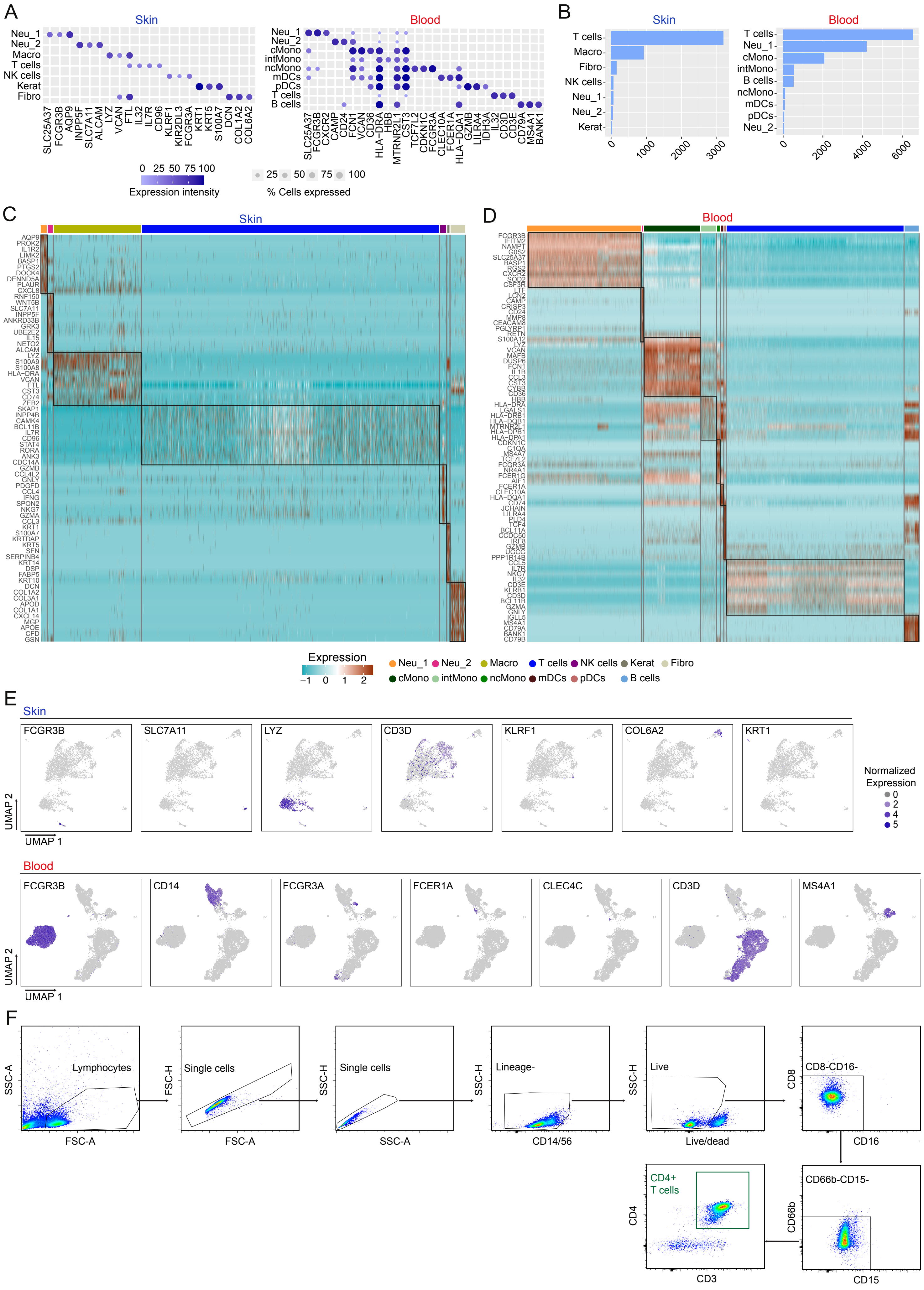

Supplement: Supplementary Figures [file mmc1.pdf]
