# Supplementary figures and images for: High-dimensional single-cell analyses reveal neutrophil heterogeneity in guttate psoriasis
Source: eBioMedicine. 2026 Feb 19;125:106172. doi: 10.1016/j.ebiom.2026.106172 (PMC12936745; doi:10.1016/j.ebiom.2026.106172)

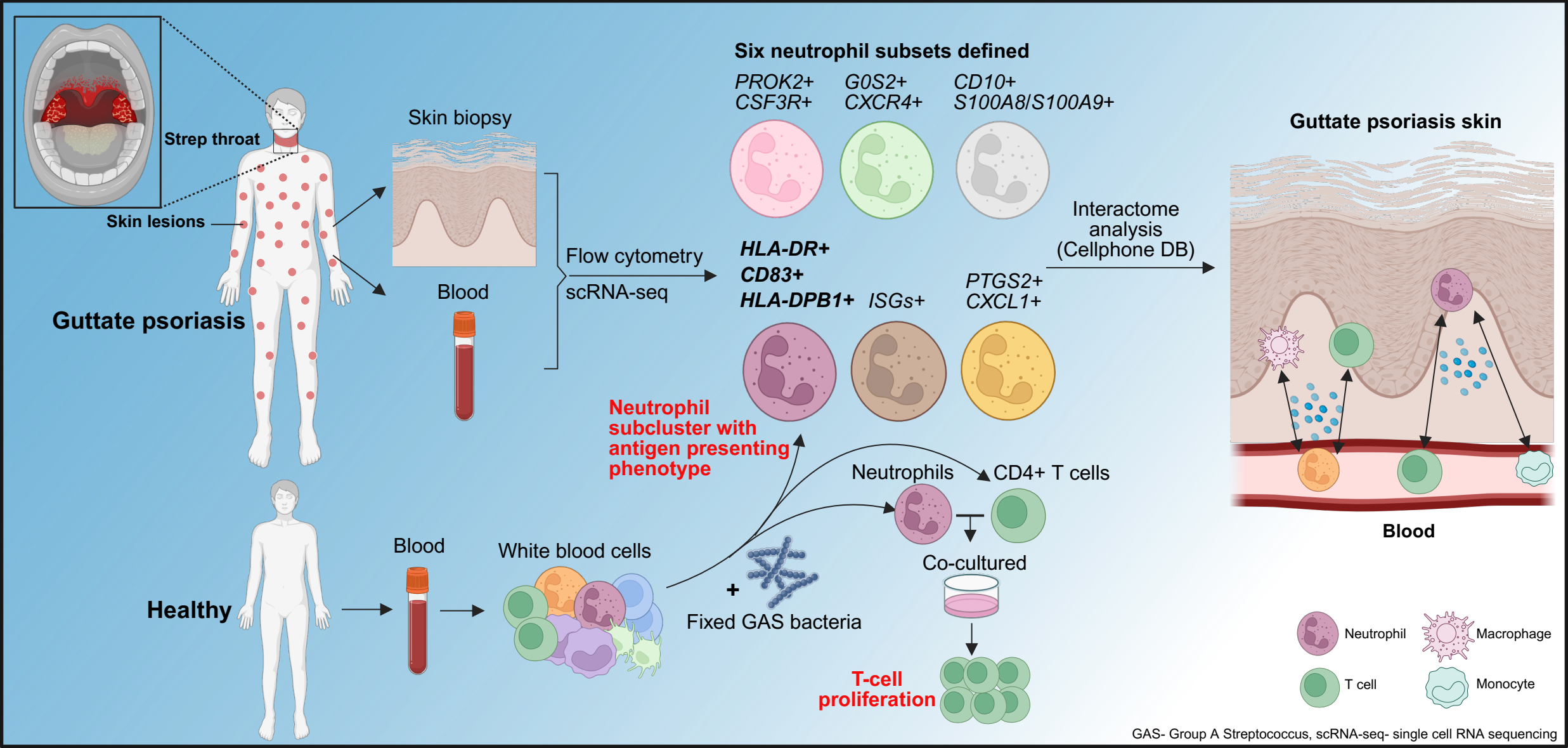

Supplement: Graphical Abstract [file mmc3.pdf]
