# Supplementary material for: High-dimensional single-cell analyses reveal neutrophil heterogeneity in guttate psoriasis
Source: eBioMedicine. 2026 Feb 19;125:106172. doi: 10.1016/j.ebiom.2026.106172 (PMC12936745; doi:10.1016/j.ebiom.2026.106172)
Supplement: Caption for supplementary material [file mmc4.pdf]

LEGENDS FOR SUPPLEMENTARY FIGURES

Figure S1. Immune and stromal cell populations in GP lesional skin and blood. (A-B) Uniform manifold approximation and projection (UMAP) analysis of all immune cells (A) and of individual immune cell populations (B) in GP skin and blood based on flow cytometry. (C-E) Median fluorescence intensity (MFI) of surface markers used to identify granulocytes (C), other myeloid cells (D), and lymphocytes (E). (F) Gating strategy for identification of neutrophils (neu) in skin and blood of patients with GP. The colour key from blue to red indicates low to high expression levels. Mono: monocytes, DCs: dendritic cells, Macro: macrophages, Lymph: lymphocytes, Eos: eosinophils.

Figure S2. **Functional similarities and differences between neutrophil populations in GP skin and blood**. (A) Gating strategy of the populations sorted from skin and blood for scRNA-seq. Neu: neutrophils. (B) UMAP visualisation of blood and skin neutrophils from each patient with GP included in the UMAP shown in **Figure 2A**. (C) Cytokine, chemokine, and chemokine receptor expression levels in the neutrophil populations found in skin and blood. The colour key from purple to red indicates low to high expression levels. The size of the dot indicates the percentage of cells expressing the indicated gene. (D) Functional scores (normalised expression) of the contents of the four granules in the six neutrophil populations in skin (blue) and blood (red). The black line indicates the median value. (E) Heatmap showing the normalised expression (Z-score) of neutrophil granule-related genes for all clusters. The numbers on top of the heatmap indicate the cluster number, the colour code on the left the type of granules, and the names on the right the genes in each cluster. (F) Differentially expressed genes upregulated (positive FC) or downregulated (negative FC) in skin neutrophil subsets relative to the corresponding blood subset. The colour key from light to dark purple indicates low to high significance (padj value).

Figure S3. Distinct neutrophil subpopulations following bacterial stimulation. (A) Gating strategy for neutrophils post-stimulation. SSC-A: Side scatter area, FSC-A: Forward scatter area, GAS: Group A Streptococcus, MOI: multiplicity of infection. Neu: neutrophils. (B) Feature plots displaying the median fluorescence intensity (MFI) of selected neutrophil markers on the UMAP shown in **Figure 3B**. The colour key from blue to red indicates low to high expression levels. (C) Bar graph showing the frequency of each neutrophil Phenograph cluster after each stimulation (presented with different colours). (D) UMAP projection of the distribution of neutrophil Phenograph clusters in the validation cohort (n=8) under the indicated

conditions. (E) Dot plot showing the frequency of CD177$^+$ neutrophils in healthy controls (HC, n=29) and patients with GP (n=21). (F) UMAP visualisation of overlaying of the average gene expression of GAS-induced "top expressed" proteins in neutrophils in **Figure 3E** on our scRNA-seq data of GP skin and blood separately. (G) UMAP visualisation of the overlaying of average gene expression of a publicly available GAS pharyngitis gene signature on our scRNA-seq data of GP skin and blood neutrophils presented as merged or individual UMAPs. Statistical analysis in (D) was done using the two-tailed non-parametric Mann-Whitney U test. ** indicate $p<0.01$.

Figure S4. **Transcriptomic and functional differences between the neutrophil subsets of healthy donors and patients with GP or ARDS.** (A) UMAP visualisation of blood neutrophils from each individual included in the UMAP shown in **Figure 4A**. (B) Top differentially expressed genes upregulated in GP neutrophil subsets (positive FC) or downregulated (negative FC) relative to the corresponding subset in healthy controls. The colour key from light to dark purple indicates low to high significance (padj value). (C) Functional scores (normalised expression) of the contents of the four granules in the seven neutrophil populations in ARDS (brown), GP (red), and healthy controls (blue). The black line indicates the median value.

Figure S5. Immune and stromal cell populations in GP lesional skin and blood identified with scRNA-seq. (A) Dot plots showing the levels of key genes used to identify individual populations. The colour key from light to dark purple indicates low to high expression levels. The dot size indicates the percentage of cells expressing the indicated gene. (B) Cell counts of the individual populations were identified and analysed after stringent quality control of scRNA-seq data. Heatmap showing the top 10 genes expressed in each of the populations in skin (C) and blood (D) based on average log2 fold change. The colour key from turquoise to brown indicates low to high expression levels. (E) Feature plots showing the expression of canonical markers used to define each cluster in skin and blood. (F) Strategy to gate CD4$^+$ T cells in CD4$^+$ T cell-neutrophil co-culture assays. Neu: neutrophils, cMono: classical Monocytes, intMono: intermediate Monocytes, ncMono: non-classical Monocytes, Macro: macrophages, mDCs: myeloid DCs, pDCs: plasmacytoid DCs, NK: natural killer cells, Kerat: keratinocytes, Fibro: fibroblasts.
